# Efeito Cardiodepressor do Acetato de Eugenil em Coração de Roedor

**DOI:** 10.36660/abc.20190823

**Published:** 2020-11-01

**Authors:** Leisiane Pereira Marques, Samuel Santos Beserra, Danilo Roman-Campos, Antonio Nei Santana Gondim

**Affiliations:** 1 Universidade do Estado da Bahia Departamento de Educação SalvadorBA Brasil Universidade do Estado da Bahia - Departamento de Educação, Salvador, BA - Brasil; 2 Universidade Federal de São Paulo – Biofísica São PauloSP Brasil Universidade Federal de São Paulo – Biofísica, São Paulo, SP – Brasil

**Keywords:** Acetato de Eugenil, Contração Miocárdica, Syzygium Aromaticum, Ratos

## Abstract

No presente trabalho investigou-se o efeito inotrópico do acetato de eugenil (AE), bem como sua ação sobre a corrente de Ca^2+^ do tipo L (I_Ca,L_). Os experimentos de contratilidade foram realizados em átrio esquerdo isolado de cobaia exposto às concentrações crescentes da droga (1 a 5.000*μ*M). O AE reduziu a força de contração atrial (IC_50_=558±24,06*μ*M) de modo dependente de concentração. O efeito do AE sobre a I_Ca,L_ também foi avaliado em cardiomiócitos ventriculares isolados de camundongos, utilizando-se a técnica de “*patch-clamp*”. O AE apresentou um efeito inibitório (IC_50_=1.337±221*μ*M) sobre os canais de Ca^2+^ sensíveis à voltagem (Ca_V_1.2). Em conclusão, o AE apesenta efeito cardiodepressor que se deve, pelo menos em parte, à diminuição da entrada de Ca^2+^ nos cardiomiócitos.

## Introdução

As doenças cardiovasculares são um problema de saúde pública e estão entre as principais causas de óbitos no mundo.^1^ Nessa perspectiva, é crescente o interesse pela busca de novas substâncias com propriedades farmacológicas sobre o sistema cardiovascular, principalmente as de origem natural.

**Figura 1:**
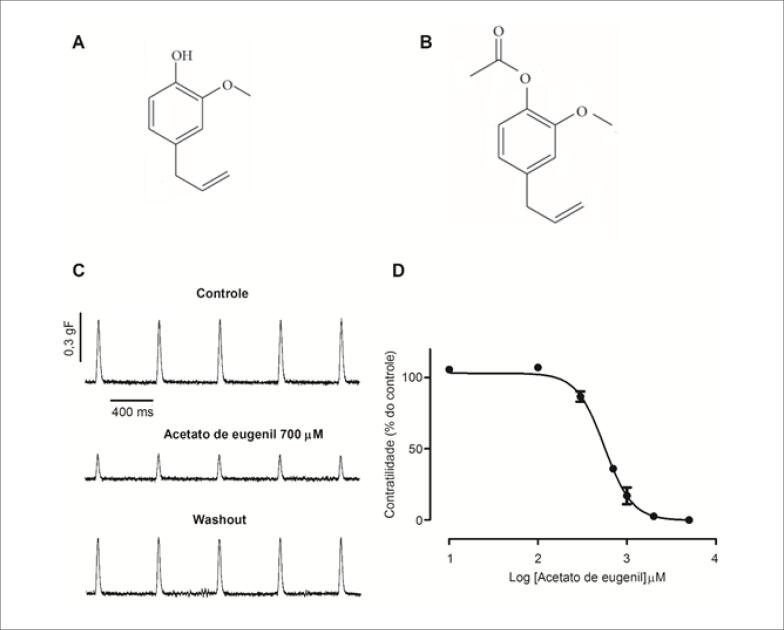
Efeito do acetato de eugenil (AE) sobre a contratilidade do miocárdio atrial. A) Estrutura do eugenol. B) Estrutura do AE. C) Traçados representativos da contratilidade atrial na situação controle, na presença de 700 *μ*M de AE, e após 10 minutos do washout. D) Curva concentração-efeito inotrópico negativo do AE (n=4).

A planta *Syzygium aromaticum* (L.) Merr. & L.M. Perry, popularmente conhecida como cravo-da-índia, é constituída por diversos compostos químicos que apresentam uma ampla gama de efeitos farmacológicos. O eugenol (Figura 1A) é o mais abundante composto bioativo encontrado no óleo essencial do cravo-da-índia, seguido pelo acetato de eugenil (AE) (Figura 1B).^2^

Estudos demonstraram que o eugenol apresenta atividade cardiodepressora em ratos^3^ e cobaias^4^ provavelmente devido à inibição da corrente de Ca^2+^ do tipo L (I_Ca,L_). Além disso, o eugenol atua como cardioprotetor.^5^

Apesar de diversos estudos abordarem as propriedades farmacológicas do eugenol sobre o coração, até o momento não existem informações sobre a ação do AE no miocárdio. Dessa forma, o presente estudo descreve, pela primeira vez, os efeitos do AE sobre a contratilidade cardíaca e sua ação inibitória sobre a I_Ca,L_.

## Métodos

### Animais

Para os experimentos de contratilidade foram utilizadas cobaias machos e fêmeas (*Cavia porcellus*, 400-600g). Para os estudos eletrofisiológicos foram utilizados camundongos machos adultos C57Bl/6J. Todos os procedimentos foram aprovados pela Comissão de Ética para Uso de Animais (CEUA) da Universidade do Estado da Bahia (licença: 03/2017).

### Protocolos Experimentais

#### Avaliação do Efeito Inotrópico do AE

O átrio esquerdo foi mantido em solução de Tyrode modificada (10 mL, 36,5±0,5°C) com a seguinte composição (em mM): 140 NaCl; 5,4 KCl; 0,5 MgCl_2_; 0,33 NaH_2_PO_4_; 11 glicose; 5 HEPES e 1,8 CaCl_2_ (pH =7,4), aerado com oxigênio (99,9%), estirado para uma tensão de 1gF e estimulado eletricamente (2Hz, 100V, 15ms). A força contrátil foi captada por um transdutor isométrico, sendo os sinais digitalizados (512Hz) e armazenados em um computador. Os átrios esquerdos foram submetidos às concentrações crescentes de AE (1-5.000*μ*M, 3 a 5 minutos).

Para fazer a solução-estoque de AE (obtido da Sigma-Aldrich) foi usado dimetil sulfóxido (DMSO).

#### Avaliação do Efeito do AE Sobre a Corrente de Cálcio Tipo L

Cardiomiócitos ventriculares de camundongos C57Bl/6J foram enzimaticamente isolados.^6^ Para medir a corrente de Ca^2+^ do tipo L (I_Ca,L_) foi utilizada a técnica de *patch-clamp*^7^^,^^8^ no modo *whole-cell voltage-clamp*. A composição da solução interna (em mM) foi: 120 CsCl, 10 HEPES, 5 EGTA, 20 TEA-Cl e 5 NaCl (pH=7,2; CsOH). O Tyrode foi usado como solução externa. As células foram mantidas em um potencial de membrara de −80 mV, e depois foram submetidas a um pré-pulso que despolarizou a membrana para −40mV (50ms). Em seguida a membrana foi despolarizada para 0 mV (300ms, 0.1Hz). A amplitude da I_Ca,L_ foi medida pela diferença entre o final do pulso teste (0mV) e o pico. As células foram expostas ao AE (10-3.000*μ*M, 2-3minutos). Os sinais foram digitalizados (5kHz) e armazenados em computador.

### Análise Estatística

Os resultados são expressos com média ± erro padrão da média e foram analisados estatisticamente empregando-se o teste “t” pareado na sua forma bicaudal (nível de significância: p<0,05).

## Resultados

### Efeito do AE Sobre a Força de Contração Miocárdica

Os traçados da Figura 1C mostram que o AE (700 *μ*M) reduziu em aproximadamente 60% a amplitude da contração atrial quando comparado com o controle. Tal efeito foi parcialmente revertido (aproximadamente 75%) após a remoção da droga, quando comparado com o controle. Na Figura 1D é possível observar a curva de concentração-efeito do AE sobre a contratilidade (n=4), o qual apresentou uma IC_50_ (concentração que induz metade do efeito máximo) de 558±24,06*μ*M e efeito máximo=100%.

### Efeito do AE Sobre a I_Ca,L_ em Cardiomiócitos Isolados

**Figura 2:**
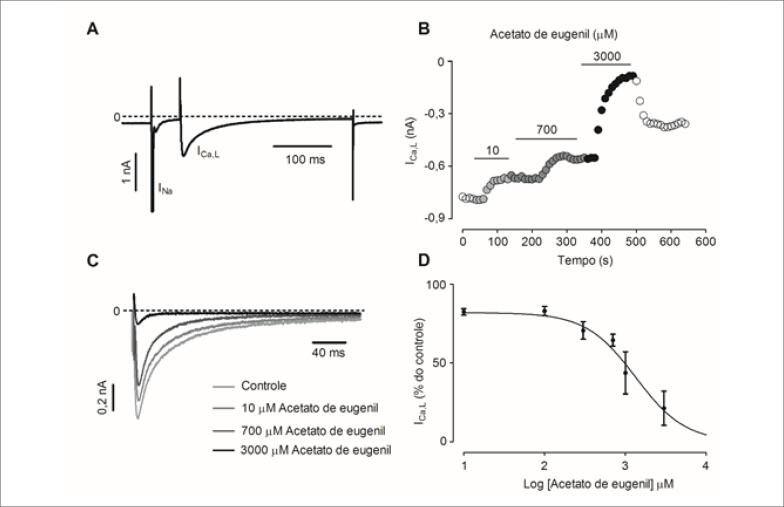
Efeito do AE sobre a I_Ca,L_. A) Corrente iônica obtida experimentalmente. B) Curso temporal do efeito do AE sobre a I_Ca,L_. C) Traçados representativos da I_Ca,L_ no controle e na presença de diferentes concentrações de AE. A linha tracejada indica zero de corrente D) Curva concentração-efeito do AE sobre a I_Ca,L_ em cardiomiócitos (n = 4).

A Figura 2A mostra o traçado representativo da I_Ca,L_ medida experimentalmente. Na figura 2B pode ser observado o curso temporal do efeito do AE sobre a amplitude da I_Ca,L_. Na Figura 2C é possível visualizar traçados representativos da I_Ca,L_ na situação controle (ausência da droga) e na presença de 10, 700 e 3.000*μ*M de AE. Na Figura 2D percebe-se que a exposição ao AE reduziu a amplitude da I_Ca,L_ de modo dependente de concentração (IC_50=_1.337±221*μ*M).

## Discussão

Os resultados aqui apresentados demonstram que o AE reduz a força de contração do músculo atrial de cobaia de modo dependente de concentração. Também foi observado que o AE inibe os canais de Ca^2+^ do tipo L (Ca_V_1.2) em cardiomiócitos.

O AE apresentou um efeito cardiodepressor sobre o inotropismo atrial. Apesar de não existir dados na literatura que demonstrem o efeito cardiodepressor do AE, dados de seus análogos, como o eugenol, estão disponíveis. O eugenol, de modo semelhante à droga aqui investigada reduz a força de contração do miocárdio ventricular de cobaia^5^ e rato,^4^ corroborando os dados aqui achados.

A força da contração do miocárdio correlaciona-se com alterações da amplitude do transiente de Ca^2+^ que é determinada pelo influxo de Ca^2+^ através dos canais de Ca^2+^ presentes no sarcolema, bem como pela quantidade de Ca^2+^ liberada pelo retículo sarcoplasmático (RS) no processo denominado de acoplamento excitação-contração. A despolarização sarcolemal leva a abertura dos canais de Ca^2+^ do tipo L durante a fase do platô do potencial de ação, o que leva a uma corrente de entrada de Ca^2+^. Esse influxo de Ca^2+^ estimula a liberação do Ca^2+^ armazenado no RS, processo conhecido como liberação de Ca^2+^ induzida por Ca^2+^, que induz na contratilidade cardíaca.^7^ Dessa forma, mecanismos que alteram o manejo intracelular de Ca^2+^ estão envolvidos na regulação da contratilidade no músculo cardíaco.

Para tentar explicar o inotropismo negativo do AE sobre o músculo cardíaco foi verificado sua ação sobre a amplitude da I_Ca,L_ em cardiomiócitos isolados. Os achados indicam que o AE reduz a amplitude da I_Ca,L,_ efeito que pode estar associado à ativação de receptores que modulam a I_Ca,L_ e/ou ao bloqueio direto desses canais. Este mecanismo pode ser o responsável pela redução da força induzida pelo AE, uma vez que leva a redução da liberação de Ca^2+^ pelo RS.

Sensch et al.,^4^ ao estudar as propriedades farmacológicas do eugenol, quimicamente semelhante ao AE, demonstraram que essa substância deprime a força de contração atrial por reduzir o influxo de Ca^2+^ nos cardiomiócitos. Nesses experimentos, foi observado que o eugenol apresenta uma IC_50_ de 127*μ*M, valor menor do que a IC_50_ do AE (1.337*μ*M). Esses dados sugerem que o eugenol é mais potente em bloquear a corrente de Ca^2+^ do que o AE.

## Conclusão

O AE apresenta efeito cardiodepressor que pode ser explicado, pelo menos em parte, pela inibição do Ca_V_1.2.
