# Supplementary material for: Efeito Cardiodepressor do Acetato de Eugenil em Coração de Roedor
Source: Arq Bras Cardiol. 2020 Nov 1;115(5):967–70. [Article in Portuguese] doi: 10.36660/abc.20190823 (PMC8452220; doi:10.36660/abc.20190823)
Supplement: Translation

# Translation: Cardiodepressive Effect of Eugenyl Acetate in Rodent Heart

**Authors:** Leisiane Pereira Marques, Samuel Santos Beserra, Danilo Roman-Campos, Antonio Nei Santana Gondim

**Affiliations:** 1 SalvadorBA Brazil Universidade do Estado da Bahia - Departamento de Educação, Salvador, BA - Brazil; 2 São PauloSP Brazil Universidade Federal de São Paulo – Biofísica, São Paulo, SP – Brazil

**Keywords:** Acectato Eugenil, Myocardial Contraction, Syzygium Aromaticum, Rats

## Introduction

Cardiovascular diseases are a public health problem being among the leading causes of death worldwide.^1^ In this perspective, there is a growing interest in the search for new substances with pharmacological actions on the cardiovascular system, including drugs of natural origin.

The *Syzygium aromaticum* (L.) Merr. & L.M. Perry, popularly known as clove, produces several chemical compounds that have a wide range of biological effects. Eugenol (Figure 1A) is the most abundant bioactive compound found in clove essential oil, followed by eugenyl acetate (EA) (Figure 1B).^2^

**Figure 1:** Effect of EA on left atrial contractility. A) Structure of eugenol. B) Structure of Eugenyl acetate (EA). C) Representative traces of left atrial contractility in the control situation (upper trace), in the presence of EA 700 *μ*M (middle trace), and 10 minutes after the washout (lower trace). D) Concentration-negative inotropic effect curve of the EA (n = 4).

Studies have shown that eugenol has cardiodepressor activity in rats^3^ and guinea pigs^4^ probably due to the inhibition of the L-type Ca^2+^ current (I_Ca,L_). In addition, eugenol acts as cardioprotector.^5^

Although several studies have adressed the pharmacological properties of eugenol on mammalian heart, so far there is no information on the action of EA on the cardiomyocytes. Thus, this study describes the effects of EA on atrial contractility and its inhibitory action on I_Ca,L_.

## Methods

### Animals

For contractility experiments guinea pigs (*Cavia porcellus*, both sexes, 400-600g) were used. For electrophysiological studies, adult male C57Bl/6J mice were used. All procedures were approved by the Ethics Committee for the Use of Animals (CEUA) of the University of the State of Bahia (license: 03/2017).

### Experimental Protocols

#### Evaluation of EA Inotropic Effect

The left atrium from guinea pig was maintained in a modified Tyrode's solution (10 mL, 36.5±0.5°C) with the following composition (in mM): 140 NaCl; 5.4 KCl; 0.5 MgCl_2_; 0.33 NaH_2_PO_4_; Glucose; 5 HEPES and 1.8 CaCl_2_ (pH=7.4), aerated with oxygen (99.9%). The atrium was stretched to attain a resting tension of 1gF and it was electrically stimulated (2Hz, 100V, 15ms). The contractile force was captured by an isometric transducer. Signals were digitized (512Hz) and stored on a computer. The left atria were subjected to increasing concentrations of EA (1-5,000*μ*M, 3 to 5 minutes for each tested concentration).

Dimethyl sulfoxide (DMSO) was used to make the stock solution of EA (obtained from Sigma-Aldrich).

#### Investigation of EA Effect on the L-type Calcium Current.

Ventricular cardiomyocytes from C57Bl/6J mice were isolated as previously described.^6^ The composition of the internal solution (in mM) was: 120 CsCl, 10 HEPES, 5 EGTA, 20 TEA-Cl, and 5 NaCl (pH=7.2; CsOH). Modified Tyrode was used as external solution. To measure L-type Ca^2+^ current (I_Ca,L_) patch-clamp technique in whole-cell voltage-clamp mode was used.^7,8^ Cells were maintained at a resting membrane potential of −80mV, then the sarcolemma was subjected to a pre-pulse to −40mV (50ms) followed by a pulse to 0 mV (300ms, 0.1Hz). The I_Ca,L_ amplitude was measured by the difference between the current at the end of the test pulse (0mV) and the peak current. The cells were exposed to EA (10-3,000 *μ*M, 2-3 minutes for each concentration). The signals were digitized (5kHz) and stored on the computer.

### Statistical Analysis

The results are expressed as mean ± standard error of the mean and were statistically analyzed using the two-tailed “t” test (significance level: p<0.05).

## Results

### EA Effect on the Left Atrial Contractility

Representative traces in Figure 1C show that EA (700 *μ*M) reduced the amplitude of atrial contraction by approximately 60% when compared to the control. This effect was partially reversed (approximately 75%) after removing the drug (washout). In Figure 1D, it is possible to observe the EA concentration-effect curve on contractility (n = 4). EA presented an IC_50_ (concentration that induces half of the maximum effect) of 558±24.06*μ*M and a maximum effect = 100%.

### EA Action on I_Ca,L_

Figure 2A depicts a representative trace of the I_Ca,L_ measured in healthy isolated cardiomyocytes. Figure 2B shows the time course of the EA effect on the I_Ca,L_. In Figure 2C, it is possible to see representative traces of I_Ca,L_ in the control (absence of the drug), and in the presence of 10, 700, and 3,000 *μ*M of EA. Figure 2D demonstrates that exposure to EA reduced the amplitude of I_Ca,L_ in a concentration-dependent manner (IC_50_=1,337±221*μ*M).

**Figure 2:** Effect of Eugenyl acetate (EA) on I_Ca,L_. A) Representative Ca^2+^ current obtained in healthy isolated cardiomyocytes. B) Time course of the EA effect on peak I_Ca,L_. C) Representative traces of I_Ca,L_ in the control and in presence of EA (10, 700, 3,000 uM). Dashed line indicates zero current. D) EA concentration-effect curve on peak I_Ca,L_ in cardiomyocytes (n = 4).

## Discussion

In our study we show that EA reduces the contraction force of guinea pig left atrial myocardium in a concentration-dependent manner. It was also observed that EA inhibits L-type Ca^2+^ channels (Ca_V_1.2) in isolated cardiomyocytes from mice.

The EA showed a cardiodepressor effect on atrial inotropism. Although there are no data in the literature that demonstrate the negative inotropic effect of AE, information from its analogues, such as eugenol, is found. Similarly to the drug investigated here, Eugenol reduced the contraction force of ventricular cardiomyocyte from guinea pig^5^ and rat,^4^ corroborating the data in our present study.

The cardiomyocyte contraction correlates with the increase in cytosolic Ca^2+^ concentration which is determined by the Ca^2+^ influx through Ca^2+^ channels present in the sarcolemma, as well by the amount of Ca^2+^ released by the sarcoplasmic reticulum (SR) in the process called excitation-contraction coupling. The membrane depolarization leads to the opening of the L-type Ca^2+^ channels mainly during the plateau phase of action potential leading to an inward Ca^2+^ current. This Ca^2+^ entry stimulates the release of Ca^2+^ stored in SR, a process known as Ca^2+^-induced Ca^2+^ release, which contributes to cardiac contractility.^7^ Thus, the mechanisms that alter the intracellular Ca^2+^ handling are involved in the control of cardiac contractility.

In order to explain the negative inotropic effect of EA on the cardiac muscle, the drug effect on the I_Ca,L_ amplitude was investigated in isolated cardiomyocytes. The findings described here show that EA reduces the peak of I_Ca,L_. This effect may be associated with the activation of receptors that modulate I_Ca,L_, and/or direct action of EA on Ca_V_1.2. The reduction of I_Ca,L_ is able to explain the decrease of contractility induced by EA, since it would lead to a reduction in the release of Ca^2+^ from SR. Although additional mechanism may also be involved.

Sensch et al.,^4^ studying the pharmacological properties of EA analogue eugenol, demonstrated that this substance depresses the force of atrial contraction by reduction Ca^2+^ influx in cardiomyocytes. In these experiments, it was observed that eugenol has an IC_50_ of 127*μ*M, a value lower than the IC_50_ observed for AE (1,337*μ*M). These data suggest that blocking effect of eugenol on Ca^2+^ channels is more potent than EA.

## Conclusion

Eugenyl acetate has a cardiodepressor effect that can be explained, at least in part, by the inhibition of Ca_V_1.2.
